# Needle-free electronically controlled jet injection with corticosteroids in recalcitrant keloid scars: a retrospective study and patient survey

**DOI:** 10.1007/s10103-023-03891-2

**Published:** 2023-11-02

**Authors:** Liora Bik, Ixora Elmzoon, Albert Wolkerstorfer, Errol P. Prens, Martijn B. A. van Doorn

**Affiliations:** 1 Department of Dermatology, Erasmus Medical Centre, Rotterdam, The Netherlands; 2 Department of Dermatology, Amsterdam University Medical Center, Amsterdam, The Netherlands

**Keywords:** Scar, Keloid, Needle-free injection, Drug delivery, Corticosteroid, Efficacy

## Abstract

First-line treatment of keloids consists of intralesional needle injections with corticosteroids, but generally entails multiple painful sessions, resulting in variable clinical outcomes. Novel needle-free jet injectors may facilitate more effective and patient-friendly dermal drug delivery. Here, we evaluated the effectiveness, tolerability and patient satisfaction of intralesional triamcinolone-acetonide (TCA) treatment in recalcitrant keloids using an electronically controlled pneumatic injector (EPI). A retrospective study was conducted in recalcitrant keloid patients with a history of severe pain during needle injections who received three sessions of EPI + TCA. Outcome measures included Patient and Observer Scar Assessment Scale (POSAS), Global Aesthetic Improvement Scale (GAIS), treatment-related pain (NRS), adverse effects, and patient satisfaction (survey). Ten patients with in total 283 keloids were included. The POSAS score significantly improved at follow-up and GAIS was reported as ‘(very) improved’ for all patients. EPI + TCA was well-tolerated with a significantly lower NRS pain score compared to needle + TCA (pilot treatment). Only minor adverse effects occurred, and 90% of patients preferred EPI over needle treatment. EPI + TCA is an effective and tolerable treatment for patients with recalcitrant keloids. The minimal treatment-related pain and high patient satisfaction makes it a promising treatment for patients with needle-phobia and/or severe pain during needle injections.

**Supplementary information:**

The online version contains supplementary material available at 10.1007/s10103-023-03891-2.

## Introduction

Keloid scars or “keloids” are benign skin tumors that consist of redundant scar tissue as a result of an abnormal wound healing process. They may arise after skin injury and extend across the original boundaries of the wound area to adjacent normal skin tissue. The incidence of keloids is estimated at 5–16% in the African and Hispanic population [1], and is associated with a lower quality of life, specifically in emotional and mental well-being [2]. In addition, the underlying pathogenesis of keloids entails chronic inflammation in the reticular dermis causing debilitating clinical symptoms such as pain and pruritis [3]. 

First-line treatment for keloids consists of intralesional needle injections with corticosteroids, most commonly triamcinolone acetonide (TCA). Intralesional corticosteroid treatment is mostly repeated every 4 to 6 weeks, with response rates varying between 50 and 100% [4–6]. Besides limiting disease progression, it results in flatting and softening of the scar tissue, and reduction of pain and pruritis [7, 8]. However, conventional needle injections have several disadvantages including the need for multiple painful sessions, highly variable and operator dependent clinical outcomes, and recurrence rates of up to 50% [4]. To overcome these restraints, alternative drug delivery methods were developed to increase the tolerability and bioavailability of corticosteroid treatment, such as micro needling, laser-assisted drug delivery, iontophoresis and pneumatic jet injectons [4, 9–12].

Jet injectors are needle-free devices that deliver drugs to the skin by generating a high-velocity liquid jet stream [13, 14]. A jet injector consists of three main components: a pressure source, a nozzle and a drug reservoir [13]. Recent developments in jet injector technology have provided sterile and safe usage by operating with disposable plastic syringes and nozzles [15–17]. These electronically controlled pneumatic injectors (EPI) use compressed gas or air as driving source and operate with tunable settings (driving pressure and injection volume). This ensures tailored treatments for high-dose dermal drug delivery at different anatomical locations with varying thickness of the skin [13].

Advantages of EPI devices over conventional needle injections include their swift operation, highly controlled method of drug delivery and minimal treatment-related pain [18–20]. However, real-world studies evaluating the clinical effects of TCA treatment in keloids using novel electronically controlled EPI with tunable settings have not been reported. Therefore, we sought to evaluate the effectiveness, tolerability, and patient satisfaction of intralesional TCA treatment using EPI in patients with recalcitrant keloids.

## Materials and methods

### Study population

This retrospective study and patient survey were carried out in the Department of Dermatology, Erasmus Medical Center in Rotterdam. Patients with recalcitrant keloids that previously received intralesional EPI with TCA treatment in our outpatient clinic from October 2020 until November 2021 were included. Patients were eligible for EPI + TCA treatment when they met the following criteria: aged ≥ 16 years old; presence of ≥ 2 recalcitrant keloid scar; a history of suboptimal results after multiple intralesional TCA needle injections; needle phobia and/or severe injection-related pain from intralesional needle injections. This study was approved by the Erasmus MC ethical and research committee (MEC-2021–0661). All patients provided written informed consent for anonymous usage of medical data and clinical photographs.

### Treatment

**Fig. 1:**
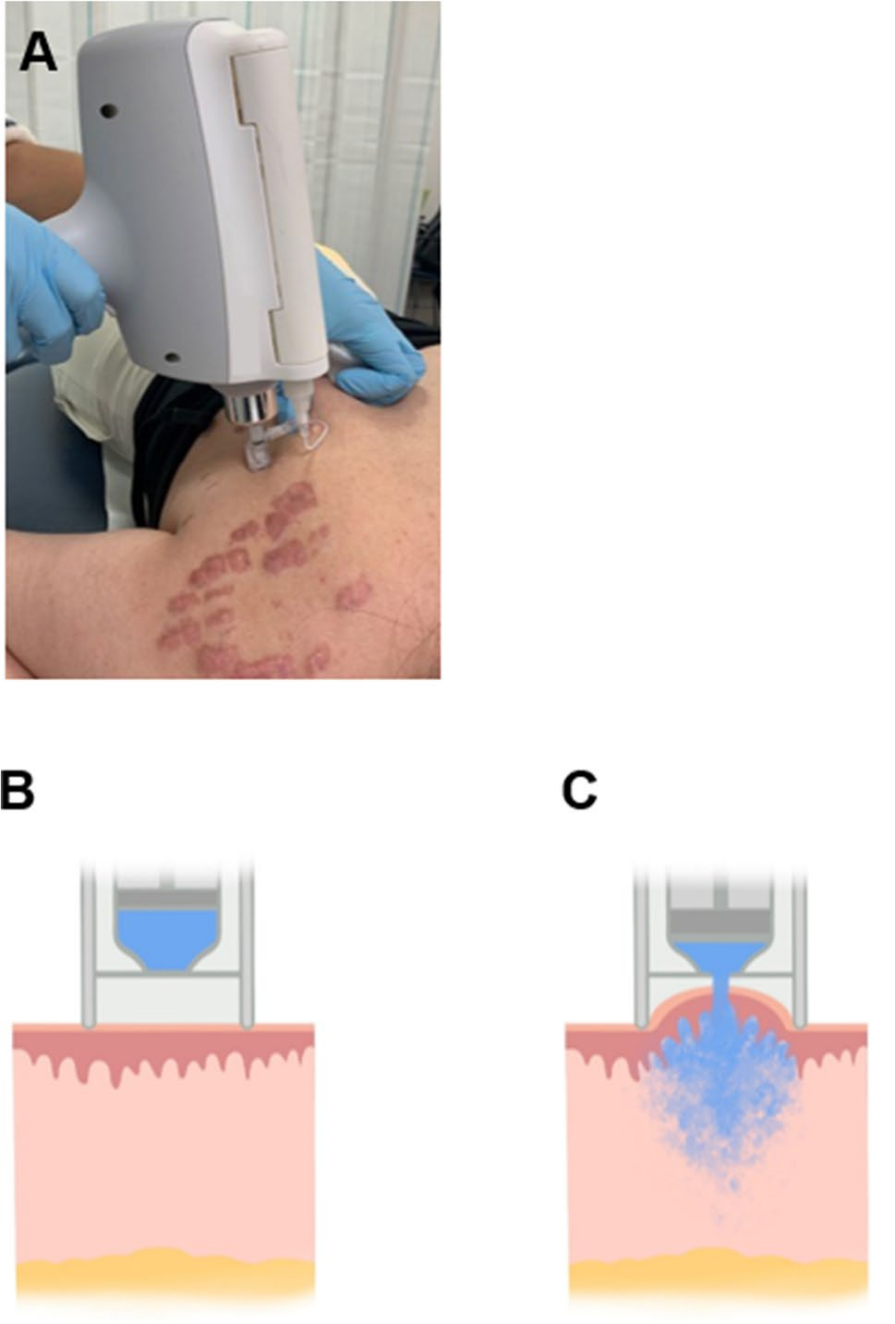
Illustrations of intralesional triamcinolone acetonide (TCA) injection with an electronically controlled pneumatic injector (EPI) device. **A**. Prior to treatment, the EPI hand piece with the injector tip is placed perpendicularly on the keloid. **B**. Schematic cross-sectioned illustration of the EPI injector tip and nozzle. The liquid container is filled with the pre-selected injection volume of 100 µL of TCA solution (blue). **C**. During injection, the high-velocity jet stream pierces the epidermis and disperses TCA solution in mid-deep dermis while inducing a visible skin papule or blanching

All patients received intralesional TCA treatments using an electronically controlled pneumatic injection device (Enerjet 2.0, PerfAction ltd, Rehovot, Israel) at an interval of 4 to 6 weeks (Fig. 1). The nozzle tip of the hand piece has an orifice diameter of 0.2 mm and generates jet stream velocities of ≤ 150 m/s with compressed air as the pressure source [21]. A pre-selected injection volume of 100 µL (device range: 50–130 µL) was used for TCA administration (10, 20 or 40 mg/ml in saline, depending on keloid thickness; Kenalog, Bristol Myers Squibb, New York, USA) with one injection per square centimeter of keloid tissue. No additional local anesthetics were required. In each patient the treatment started at a low pressure of 2 bar (device range: 2–6 bar) and was titrated up until a skin papule and/or blanching was induced (clinical endpoint), indicating successful dermal drug delivery as previously described [20]. Clinical photographs were collected at each visit.

Prior to the first treatment, treatment-related pain (NRS; range 0–10) and patient preference was determined for EPI + TCA (10–20 mg/ml) and needle + TCA (27G; 10–20 mg/ml) in an intra-patient pilot session of two similar keloids. At the next session, patients were asked to if they preferred continued intralesional TCA administration with conventional needle injections or EPI.

### Data collection and outcome measures

Data was collected from the electronic medical records. Baseline data included age, gender, Fitzpatrick skin type, number of lesions, laterality, lesion size and description, previous treatments, comorbidity and motivation for EPI treatment. The primary outcome measure was clinical effectiveness and secondary outcomes measures were tolerability and patient satisfaction.

Clinical effectiveness was assessed by comparing the POSAS 2.0 (Patient and Observer Scar Assessment Scale) at baseline and 6 weeks after three EPI + TCA treatments were administered (follow-up). The POSAS scores are given from 0 (normal skin) to 10 (worst imaginable scar) per subcategory with a maximum total score of 70 [22]. In addition, effectiveness was assessed with the GAIS (Global Aesthetic Improvement Scale), which consists of 5 degrees of esthetical clinical improvement scored by the treating physician: 1: exceptional improvement (excellent corrective result), 2: very improved patient (marked as improvement of appearance but not completely optimal), 3: improved patient (improvement of appearance better compared with the initial condition but additional treatment is advised), 4: unaltered patient (the appearance substantially remains the same compared with the original condition) and 5: worsened patient (the appearance has worsened compared with the original condition). Tolerability was assessed by evaluating treatment-related pain scores (NRS; range 0–10) and adverse effects, documented by the treating physician at every visit. If skin atrophy was reported by the physician, independent evaluation of clinical photographs was performed by two researchers (L.B. & I.E.) to assess the exact number of affected keloids. Discrepancy was resolved by discussion. Treatment satisfaction was evaluated by conducting a patient survey at follow-up on: a) overall treatment satisfaction, b) patient preferences for EPI + TCA versus needle + TCA treatment, c) recommendation of EPI + TCA treatment to others, and d) disturbance of EPI noise during injection. The electronic survey was conducted using LimeSurvey Version 2.06 (LimeSurvey GmbH, Hamburg, Germany).

### Statistical analyses

Statistical analysis was performed with SPSS 25.0 (IBM, Armonk, NY, USA). Data was presented as mean and SD ( ±). A paired *t*-test was used for the change of the POSAS scores and treatment-related pain scores. A *p*-value of ≤ 0.05 was considered as statistically significant.

## Results

### Patient characteristics

Ten patients (6/10 female) with 283 small and large keloids met the eligibility criteria and received EPI + TCA treatment. Baseline characteristics of patients and keloids are shown in Table 1. Patients visited the outpatient clinic for a pilot treatment and three full treatment sessions with an interval of 6.9 ± 3.4 weeks and a follow-up period of 5.6 ± 1.4 weeks. EPI injections were performed with 100 µL and a pressure ranging from 2.7 to 4.8 bar, depending on the appearance of the defined clinical endpoint (skin papule and/or blanching) immediately after injection. In 70% (21/30) of the treatments, a concentration of 20 mg/ml TCA was used. The remaining treatments were performed with a TCA concentration of either 10 mg/ml (10/30) or 40 mg/ml (3/30).

**Table 1:** Baseline characteristics

Characteristic	*N* (%), *n* = 10
Gender	
Female	6 (60%)
Age (mean, SD)	25.5 ± 10.5
Fitzpatrick skin type	
1–2	3 (30%)
3–4	5 (50%)
5–6	2 (20%)
Number of lesions	
1–10	4 (40%)
11–30	2 (20%)
31–70	4 (40%)
Anatomical location	
Head/neck	1 (10%)
Shoulders/back	4 (40%)
Thorax and shoulders/back or legs	5 (50%)
Etiology	
Trauma	1 (10%)
Acne	6 (60%)
Other/mixed causes	3 (30%)
Previous treatments*	
Multiple needle + TCA (+ bleomycin) treatments	10 (100%)
Vascular/ablative laser treatment	4 (40%)
Cryotherapy	2 (20%)
Shave excision	2 (20%)
Silicon sheeting	2 (20%)
Motivation for EPI + TCA treatment*	
Suboptimal or no results after previous treatments	10 (100%)
Severe pain during needle injections	8 (80%)
Needle-phobia	1 (10%)

Needle: needle-syringe injection

EPI: electronically controlled pneumatic injector

TCA: triamcinolone acetonide

*Multiple combinations possible

### Clinical effectiveness

**Fig. 2:**
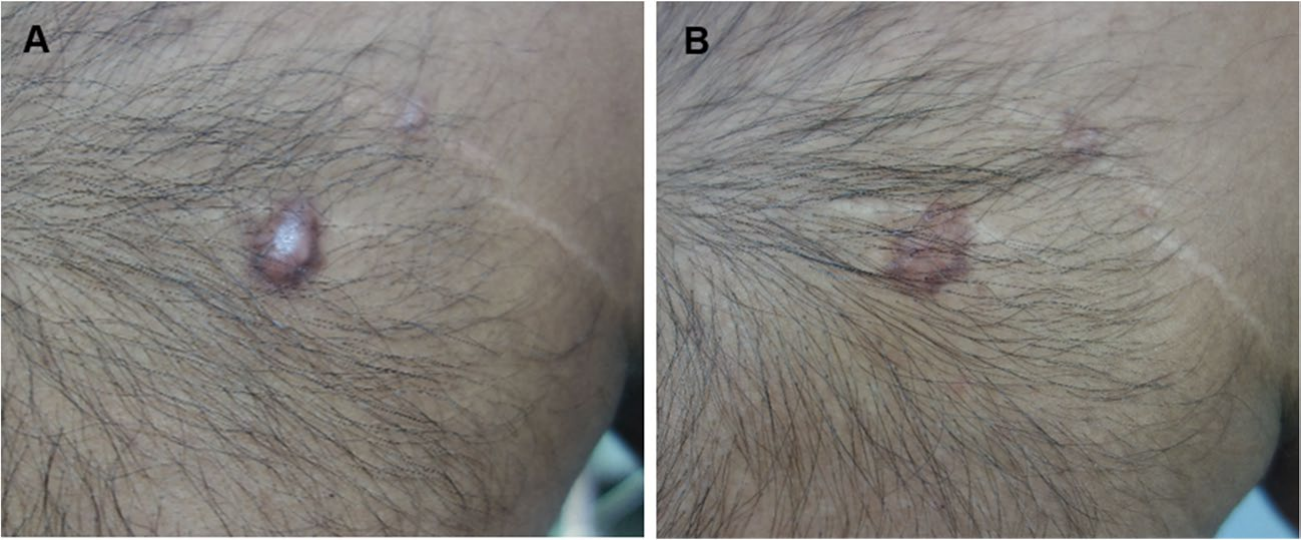
A 34-year-old man with a keloid on the chest that showed complete flattening after three intralesional triamcinolone acetonide treatments administered with an electronically controlled pneumatic injector. **A**) Keloid on the chest before treatment and **B**) after three treatments

**Fig. 3:**
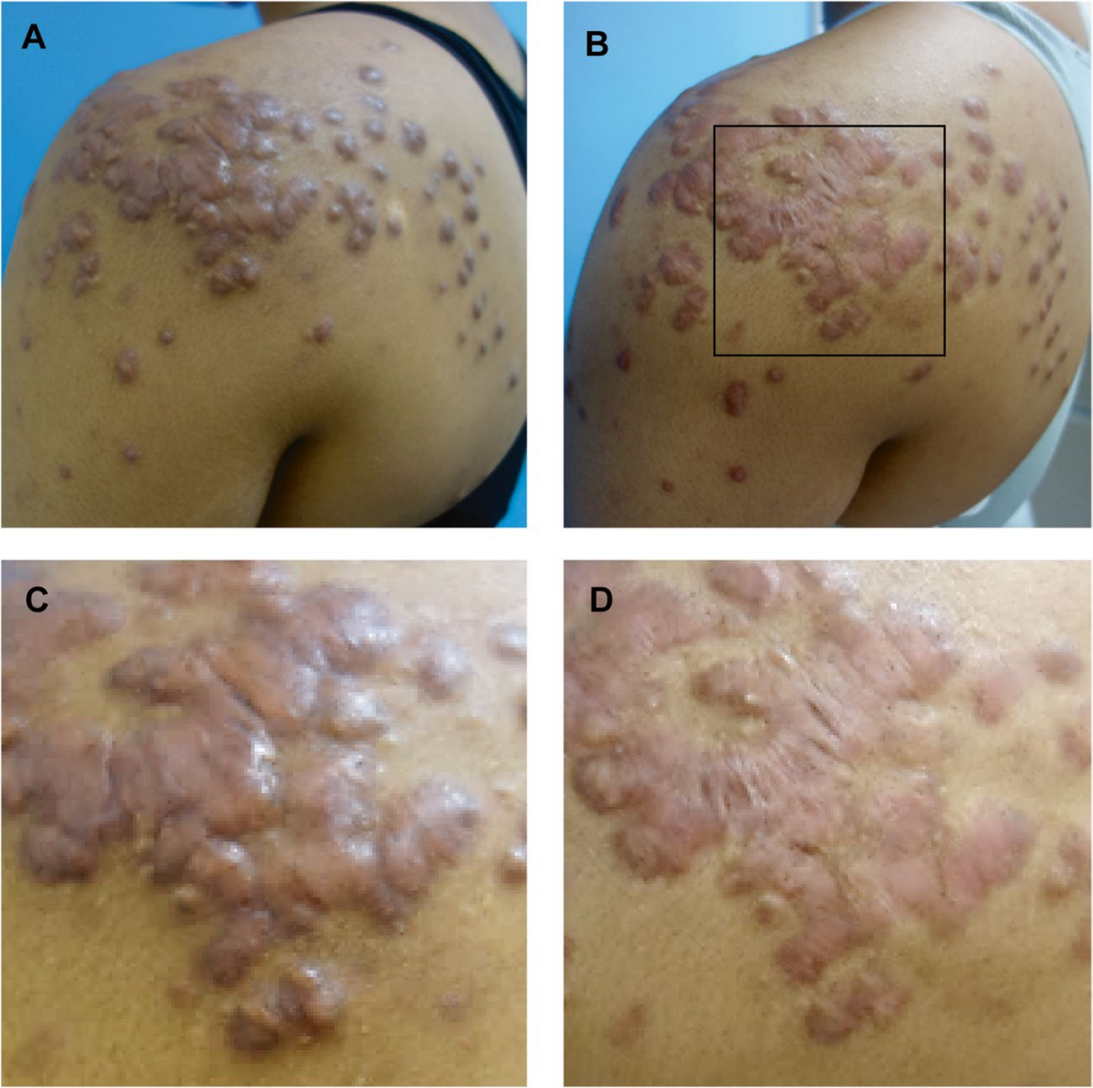
A 17-year-old woman with severe post-acne keloids and hyperpigmentation on the left back and shoulder. The keloids showed flattening, lighting and relief of pain symptoms after three intralesional triamcinolone acetonide treatments administered with an electronically controlled pneumatic injector. **A**) Clinical overview photography of the keloids prior to treatment and **B**) after three treatments. **C**) Close-up clinical photography of the keloids before treatment and **D**) after three treatments. Black square represents area of close-up

**Fig. 4:**
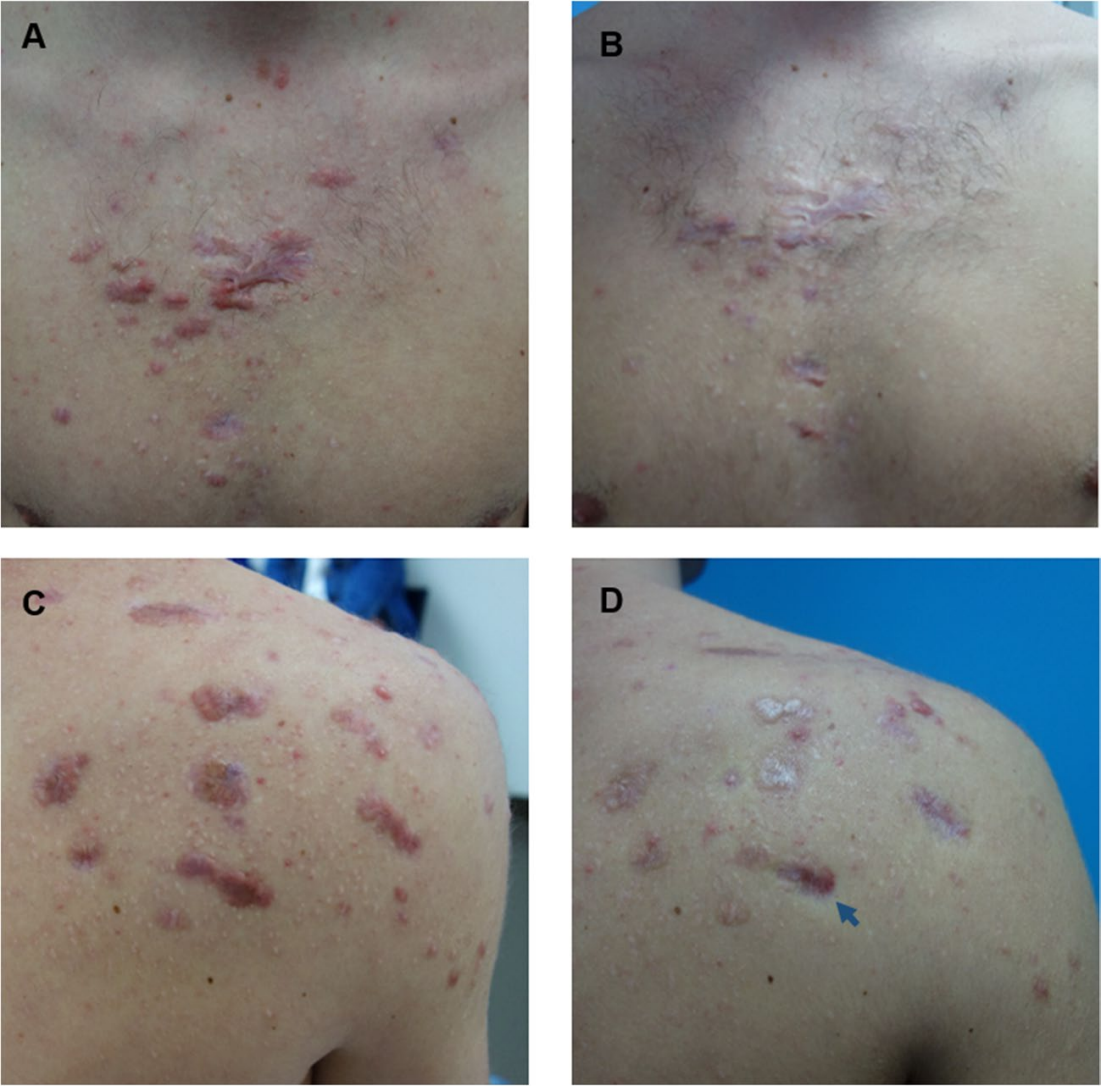
A 21-year-old man with erythematous post-acne keloids on the chest and right shoulder. The keloids showed flattening and lightening after three intralesional triamcinolone acetonide treatments administered with an electronically controlled pneumatic injector. **A**) Erythematous post-acne keloids on the chest before treatment and **B**) after three treatments. **C**) Erythematous post-acne keloids on the right shoulder before treatment and **D**) after three treatments. The blue arrow shows a representative keloid with mild skin atrophy of the adjunctive healthy skin tissue. Atrophy was not present in the rest of the treated skin lesions

**Fig. 5:**
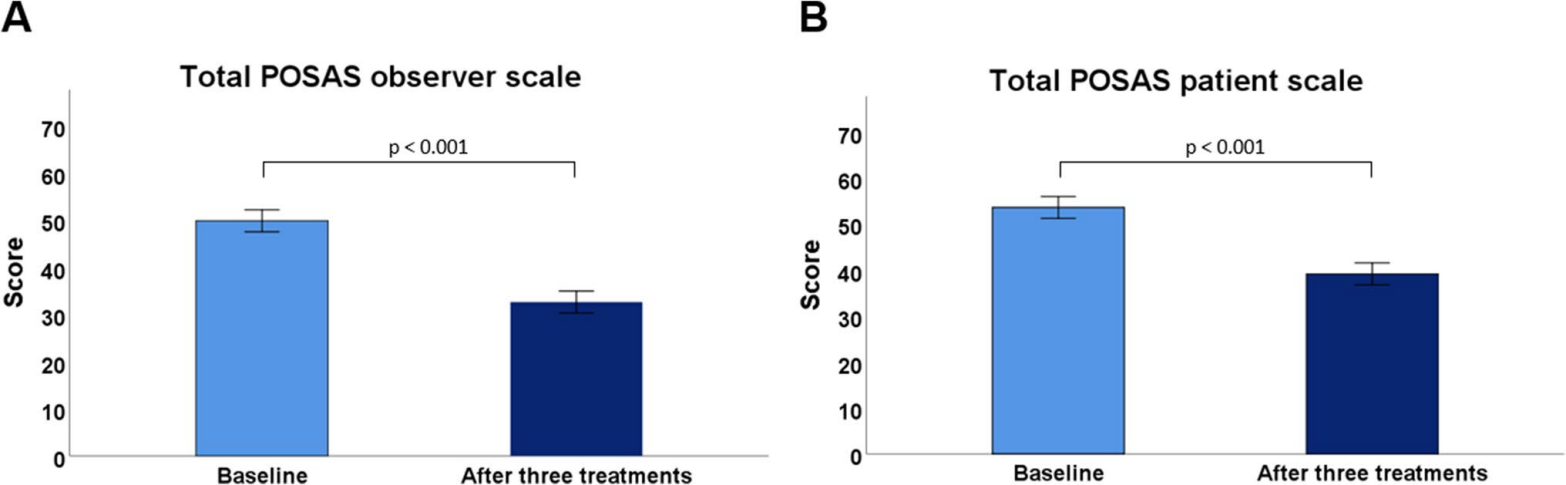
Clinical effectiveness assessed with POSAS at baseline and after three consecutive treatments of intralesional triamcinolone acetonide administered with an electronically controlled pneumatic injector (EPI + TCA) in keloids. **A**) The total POSAS “observer” scale score showed a significant improvement in keloid appearance reported by the treating physician. **B**. The total POSAS “patient” scale score showed a significant improvement in self-reported keloid quality and keloid-related symptoms. POSAS: Patient and Observer Scar Assessment Scale

Clinical improvement was observed in all patients for both POSAS and GAIS (Table 2; Figs. 2, 3, 4). The POSAS “observer” scale showed a significant improvement in keloid appearance from 49.9 ± 6.6 to 32.7 ± 9.5 (*p* < 0.001, Fig. 5-A). The clinical improvement was reported for all subcategories including vascularity, pigmentation, thickness, relief, pliability, surface area and the overall assessment. The POSAS “patient” scale showed a significant improvement in keloid quality and keloid-related symptoms with a significant decrease of 53.6 ± 9.5 to 39.2 ± 7.5 (*p* < 0.001, Fig. 5-B). In addition, the overall esthetical clinical effect assessed with GAIS was reported as “improved” in 6/10 patients and ‘very improved’ in 4/10 patients.

**Table 2:** Clinical improvement assessment using POSAS and GAIS

		Baseline, *n* = 10	EPI + TCA (3x), *n* = 10	
	*Improvement (%)*	*Mean* ± *SD*	*Mean* ± *SD*	*P-value*
Total POSAS observer scale	34.5	49.9 ± 6.6	32.7 ± 9.5	< 0.001
*Subcategories*				
Vascularity		8.1 ± 1.4	5.4 ± 3.0	
Pigmentation		4.2 ± 2.7	3.2 ± 1.7	
Thickness		8.2 ± 0.9	4.9 ± 1.9	
Relief		7.0 ± 1.9	4.8 ± 1.5	
Pliability		7.6 ± 1.4	4.3 ± 1.5	
Surface area		6.9 ± 1.2	5.0 ± 1.8	
Overall assessment		7.9 ± 0.7	5.1 ± 1.4	
Total POSAS patient scale	26.9	53.6 ± 9.5	39.2 ± 7.5	< 0.001
*Subcategories*				
Pain		4.2 ± 3.0	2.0 ± 1.3	
Itching		6.6 ± 2.9	4.2 ± 2.1	
Color		8.7 ± 1.1	7.5 ± 0.7	
Stiffness		8.0 ± 1.9	6.5 ± 1.7	
Thickness		8.7 ± 1.5	6.4 ± 2.1	
Irregularity		8.7 ± 1.6	6.1 ± 2.1	
Overall opinion		8.7 ± 1.1	6.5 ± 1.8	
			EPI + TCA (3x), n = 10 (%)	
GAIS				
Exceptional improved			.	
Very improved			4 (40%)	
Improved			6 (60%)	
Unaltered			.	
Worsened			.	

POSAS: Patient and Observer Scar Assessment Scale 2.0

GAIS: Global Aesthetic Improvement Scale

### Tolerability

Treatment-related pain and adverse effects were evaluated for assessment of tolerability (Table 3 and 4). At the initial pilot treatment, EPI + TCA resulted in significant lower treatment-related NRS pain scores than needle + TCA (4.3 ± 1.9 vs. 6.1 ± 1.9; *p* = 0.019). In addition, treatment-related pain of the following EPI + TCA treatments was reported in 22/30 treatment sessions with a NRS score of 3.8 ± 1.5 (Table 3).

**Table 3:** Treatment-related pain

		Needle + TCA, *n* = 10	EPI + TCA, *n* = 10	
Treatment-related pain (NRS)	*Difference (%)*	*Mean* ± *SD*	*Mean* ± *SD*	*P-value*
Pilot treatment*	29.5	6.1 ± 1.9	4.3 ± 1.9	0.019
*Three EPI* + TCA treatments**			3.8 ± 1.5	

Needle: needle-syringe injection

EPI: electronically controlled pneumatic injector

TCA: triamcinolone acetonide

NRS: Numerical Rating Scale (range 0–10)

^*^Intra-patient comparison of needle + TCA and EPI + TCA

^**^Treatment-related pain (NRS) reported in 22 out of a total of 30 EPI + TCA treatments

**Table 4:** Adverse effects

	*N* (%), *n* = 10
Minor adverse effects	
Transient pain, sensitivity or burning	5 (50%)
Itching	2 (20%)
Superficial deposition of TCA crystals (white dots)	1 (10%)
Hematoma	1 (10%)
Mild skin atrophy	4 (40%)* / 11 (4%)**
Infection	0 (0%)
Telangiectasia	0 (0%)

^*^Four out of 10 patients (40%) experiences mild skin atrophy in at least one of their treated keloids after the third treatment

^**^Eleven out of in total 283 treated keloids (4%) showed skin atrophy after the third treatment

TCA: triamcinolone acetonide

Reported adverse effects were mild and included transient pain, skin sensitivity or a burning sensation in 50% of the patients (5/10), local itching in 20% (2/10) and a small hematoma in a single keloid in one of the patients directly after treatment at the injection site (Table 4). In one patient, 1–2mm white dots were visible in the treated keloids, which was most likely related to superficial deposition of TCA crystals, and spontaneously resolved at follow-up. Mild local skin atrophy of adjacent healthy skin was observed in 11 out of 283 keloids (4%) after the third treatment, which occurred in four patients (Fig. 4-D). No severe adverse effects were reported.

### Patient satisfaction survey

A patient survey was conducted on the treatment satisfaction. All patients (10/10) were satisfied with the EPI + TCA treatment, and 90% (9/10) would recommend this type of treatment to others (Table 5). TCA administration with EPI was preferred over conventional needle-syringe injections by 90% (9/10) of the patients. The preference for EPI administration was mostly based on the lower degree of pain during EPI treatment, reported by 80% (8/10) of the patients. However, the EPI device generates a small air-pumping noise during injection, which was experienced as “scary” by the patient who would not recommend this treatment to others. The other patients (9/10) did not consider the EPI generated noise as disturbing in any way.

**Table 5:** Treatment satisfaction survey

	*N* (%), *n* = 10
Satisfied with EPI + TCA treatment?	
(Strongly) agree	10 (100%)
Neutral	.
(Strongly) disagree	.
Not applicable	.
Recommendation of EPI + TCA treatment to others?	
Yes	9 (90%)
No	.
Unknown	1 (10%)
Treatment preference?	
Needle + TCA	.
EPI + TCA	9 (90%)
No preference	1 (10%)
Reason for preference of EPI + TCA over needle + TCA?*	
Less painful	8 (80%)
Less time	3 (30%)
Better results	5 (50%)
Disturbed by noise of EPI device?	
Yes	1 (10%)
No	9 (90%)

Needle: needle-syringe injection

EPI: electronically controlled pneumatic injector

TCA: triamcinolone acetonide

^*^Multiple answer question

## Discussion

To our knowledge, this is the first study that evaluated the real-world effectiveness, tolerability and patient satisfaction of intralesional TCA treatment using a needle-free electronically controlled pneumatic injector in patients with recalcitrant keloids. We retrospectively observed a significant clinical improvement measured with POSAS and GAIS after three consecutive treatment sessions. Overall, EPI treatment was well-tolerated with low reported treatment-related pain scores, and occurrence of only minor adverse effects. In addition, patients were highly satisfied with the EPI + TCA treatment and would recommend this treatment to others, mainly because of the mild EPI-related injection pain.

To our knowledge, no previous studies investigated TCA injections in patients with similar recalcitrant keloids, while evaluating the clinical effectiveness with the POSAS. Nor et al., however, reported an improvement in the total POSAS “observer” score of 34.7% following three intralesional needle + TCA (40 mg/ml) treatments in previously untreated keloids with an interval of 4 weeks [23]. In addition, Wang et al. reported a 45.8% and 45.6% improvement in respectively the POSAS “patient” and “observer” scale after three intralesional needle + TCA (10 mg/ml) treatments, although at a shorter interval of 2 weeks [24]. These studies, however, used conventional needle injections to deliver TCA and did not include an evaluation of treatment-related pain. In addition, we used the GAIS score to confirm the clinical improvement observed with the POSAS. All treated keloids were considered “improved’ or “very improved” at follow-up. None of the treated keloids were scored ‘excellent improved’, which could be expected since even successful treatments will leave a flat scar with at least some discoloration and altered skin texture [25]. We are convinced that the clinical improvement we observed in this retrospective study is meaningful to patients. However, the outcome measures we used in this study have not been sufficiently validated for keloid scars.

Tolerability was assessed by evaluating the treatment-related NRS pain scores and adverse effects. We found that EPI + TCA was well-tolerated with a NRS pain score of 4.3 ± 1.9, which was 29.5% lower compared to needle + TCA injection. Studies evaluating disease or treatment-related pain mainly used a minimal clinical important difference (MCID) of NRS pain scores of ≥ 30% improvement, corresponding to the reported pain score reduction for EPI in this study [26, 27]. Previous studies reported minimal patient discomfort for EPI treatments with TCA + 5-fluorouracil in keloids or hyaluronic acid in wrinkles and acne scars [18, 28, 29]. Levenberg et al. investigated intralesional TCA + 5-fluorouracil treatment administered by EPI (same device) in keloids using pressure levels between 3.5 to 5.7 bar and an injection volume of 100 µL [18]. They reported a treatment-related NRS pain score of 2.0 ± 1.0, which is lower compared to the NRS pain score of 3.8 ± 1.5 in our study when using lower pressures ranging from 2.7 to 4.8 bar. This discrepancy could be explained by the use of 0.1% lidocaine that was added to the TCA + 5-fluorouracil mixture in the study by Levenberg et al., and/or by racial, ethnic or cultural differences of the patient groups that might experience pain in a different way [18, 30]. Furthermore, 80% of our patients reported severe pain during previous needle + TCA treatments as motivation for choosing EPI + TCA, which could have led to the selection of patients with a higher sensitivity for pain.

Adverse effect monitoring showed mild adverse effects that resolved spontaneously within 1–2 days after EPI + TCA treatment. These minor adverse effects included mild pain, sensitivity, burning sensation, itching, superficial deposition of TCA crystals (white dots) and small hematoma at the injection site. The mild atrophy observed in 11 out of 283 treated keloids occurred in four patients and could be due to unwanted dispersion of the jet stream to adjacent healthy skin, directly visible as formation of a skin papule or blanching, or delayed diffusion of the TCA solution. This is in the range of the reported skin atrophy rate after conventional TCA needle injections of 23.5–60% of keloid patients [23, 31–33]. A potential solution to prevent atrophy could be to reduce the injection volume or pressure level during the treatment of nearly flattened keloids to reduce the diffusion range, and consider lowering the TCA concentration.

In addition to effectiveness and tolerability, we evaluated the patient satisfaction with EPI + TCA treatment at follow-up. In line with the clinical improvement, all patients (100%) were satisfied with this treatment and 90% preferred TCA delivery by EPI above conventional needle injections, probably due to low treatment-related pain. These results support the use of EPI + TCA in clinical practice, especially for patients with multiple keloids that experience needle-phobia or suffered from severe pain during previous multiple intralesional TCA needle injections.

TCA delivery by EPI allows for a deep, controlled penetration throughout the fibrotic keloid scars in a standardized manner. The device settings can be adjusted during the treatment to achieve the desired clinical endpoints (skin papule and/or blanching) and high-dose dermal drug delivery. In addition, EPI generates a high-velocity jet stream of ≤ 150 m/s that penetrates the skin and disrupts excessive collagen bundles and fibrotic strands under high pressure, which by itself may improve some aspects of the keloid tissue [18]. On the other hand, the high purchase costs of the device and disposable plastic nozzles could be an obstacle for its broad application in clinical practice [20].

An important strength of this study is that it evaluates for the first time the real-world effectiveness of EPI + TCA treatment in keloids using two standardized scoring scales (POSAS and GAIS). We further evaluated treatment-related NRS pain scores of EPI + TCA relative to needle injection, and conducted a treatment satisfaction survey.

Limitations of this study include the uncontrolled retrospective study design, small sample size and a variable treatment interval of 6.9 ± 3.4 weeks mainly caused by reduced capacity of our outpatient clinic due to the COVID-19 pandemic, and a short follow-up period of 5.6 weeks precluding the assessment of recurrences.

## Conclusion

In conclusion, we found that EPI is an effective and tolerable dermal drug delivery technique for intralesional TCA administration in recalcitrant keloids. The high patient satisfaction and minimal treatment-related discomfort makes it an attractive treatment option for keloid patients, especially for those with needle-phobia and/or extreme pain during conventional needle therapy. Future high-quality prospective controlled studies investigating EPI with TCA in patients with recalcitrant keloid scars are warranted to confirm our findings.

### Supplementary information

Below is the link to the electronic supplementary material.

Supplementary file1 (PDF 498 KB)

## Abbreviations

EPI: Electronically controlled pneumatic injector
TCA: Triamcinolone acetonide
Needle: Needle-syringe injection
POSAS: Patient and observer scar assessment scale
GAIS: Global aesthetic improvement scale
NRS: Numerical rating scale
