# Supplementary material for: Needle-free electronically controlled jet injection with corticosteroids in recalcitrant keloid scars: a retrospective study and patient survey
Source: Lasers Med Sci. 2023 Nov 2;38(1):250. doi: 10.1007/s10103-023-03891-2 (PMC10622365; doi:10.1007/s10103-023-03891-2)
Supplement: Supplementary file 1 — Supplementary file1 (PDF 498 KB) [file 10103_2023_3891_MOESM1_ESM.pdf]

	Before treatment	After 3 treatments
30-year old patient with keloids on the upper left leg	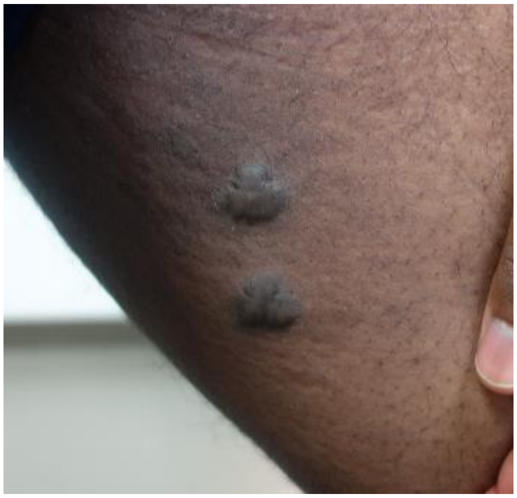	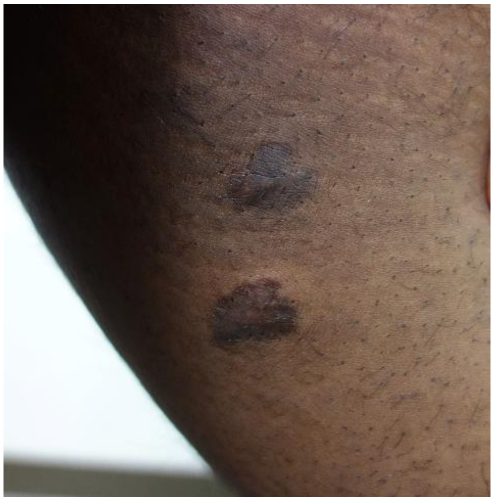
20-year old patient with keloids on the jaw line	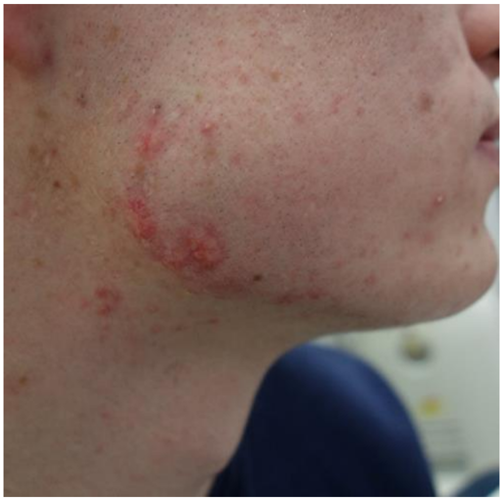	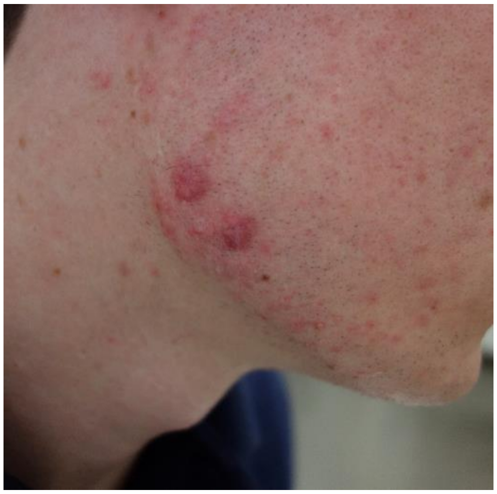
33-year old patient with keloid on the right shoulder	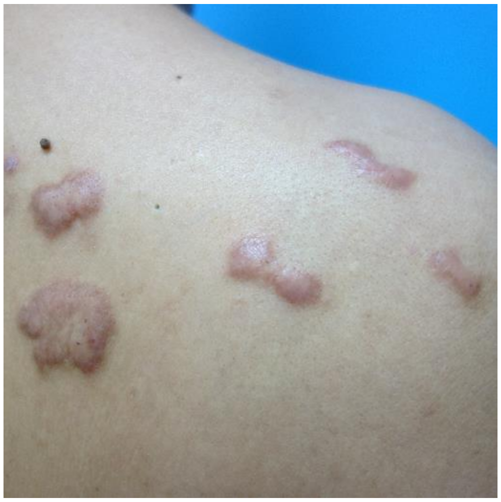	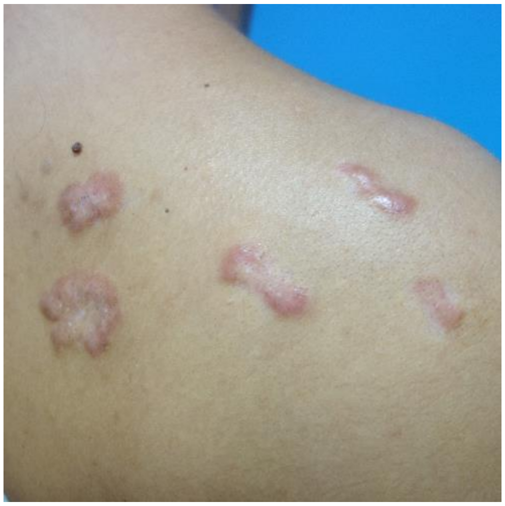
